# Predictors of treatment resistant schizophrenia: a systematic review of prospective observational studies

**DOI:** 10.1017/S0033291719002083

**Published:** 2021-01

**Authors:** S. E. Smart, A. P. Kępińska, R. M. Murray, J. H. MacCabe

**Affiliations:** Department of Psychosis Studies, Institute of Psychiatry, Psychology, & Neuroscience, King's College London, 16 de Crespigny Park, London, SE5 8AF, UK

**Keywords:** First episode, longitudinal, prediction, psychosis, schizophrenia, treatment resistant

## Abstract

Treatment-resistant schizophrenia, affecting approximately 20–30% of patients with schizophrenia, has a high burden both for patients and healthcare services. There is a need to identify treatment resistance earlier in the course of the illness, in order that effective treatment, such as clozapine, can be offered promptly. We conducted a systemic literature review of prospective longitudinal studies with the aim of identifying predictors of treatment-resistant schizophrenia from the first episode. From the 545 results screened, we identified 12 published studies where data at the first episode was used to predict treatment resistance. Younger age of onset was the most consistent predictor of treatment resistance. We discuss the gaps in the literature and how future prediction models can identify predictors of treatment response more robustly.

## Predictors of treatment-resistant schizophrenia: A systematic review of prospective observational studies

For approximately a third of patients with schizophrenia, standard antipsychotic medications do not adequately alleviate their psychotic symptoms (Conley and Kelly, 2001). This subgroup is termed treatment-resistant schizophrenia (TRS). The most common clinical and research criteria used for TRS is the failure to respond to two trials of non-clozapine antipsychotics, of adequate dose and duration (Suzuki *et al*., 2011; Howes *et al*., 2017).

Patients with TRS have higher rates of unemployment, worse quality of life, and poorer social and occupational functioning than people who respond to treatment (Iasevoli *et al*., 2016). Researchers have estimated that the direct healthcare costs for TRS in the US is 3–11-fold higher than for the schizophrenia population as a whole, with multiple hospitalisations accounting for a large proportion of this cost (Kennedy *et al*., 2014). In England, 25–50% of the National Health Service's (NHS) £11.8 billion mental health budget is allocated to schizophrenia services and TRS is thought to contribute a large proportion of these costs (Andrews *et al*., 2012; Killaspy *et al*., 2013).

Clozapine is the only antipsychotic recommended for TRS and is more effective than other antipsychotics in alleviating psychotic symptoms in patients with TRS (Kane *et al*., 1988; Siskind *et al*., 2016; Taylor, 2017). However, owing to its adverse effects, clozapine is only licenced in the UK (NICE, 2014) and most other developed countries (Warnez and Alessi-Severini, 2014) as a third-line treatment. Nevertheless, evidence suggests that TRS is often not recognised promptly, and that clozapine is offered after a delay of some years or not at all. According to treatment guidelines, the earliest that patients can be diagnosed with TRS, and prescribed clozapine, is 12 weeks after commencing antipsychotic treatment; however, Howes *et al*. (2012) report an average delay of 3.9 years, suggesting that there is considerable scope to shorten this period of inadequate treatment. Furthermore, patients with a shorter delay before clozapine initiation show a better symptomatic response to clozapine (Yoshimura *et al*., 2017).

Thus, there is a need to identify patients-who are likely to develop TRS-earlier in the course of their illness and expedite their access to specialist treatment; this may require moving beyond the current definition of TRS towards criteria based upon predictors and biomarkers, which quantify a patient's risk of developing TRS. If predictors of TRS can be identified, they may be useful in three ways: firstly, to identify TRS patients earlier in treatment so that they can be offered effective treatments earlier; secondly, to identify patients for clinical trials of interventions for TRS; and thirdly, to improve our understanding of the aetiology of TRS.

We present a comprehensive systematic review of all prospective observational studies in schizophrenia populations, which report baseline predictors of TRS. We focused solely on prospective observational studies to draw clearer conclusions regarding the causal relationship between predictors and TRS in naturalistic settings over a long follow-up, and because only longitudinal studies can identify risk factors at first episode that might predict TRS.

## Method

### Inclusion/exclusion criteria

Studies were included if they met the following inclusion criteria: (1) participants were diagnosed with schizophrenia, schizophreniform disorder, schizoaffective disorder, and/or a psychotic disorder; we did not exclude studies that *also* included affective disorders or substance-induced psychosis, given the diagnostic uncertainly around the first episode of psychosis; (2) participants were followed from the first episode or first treatment with antipsychotics; (3) the majority of participants were aged between 16 and 64 at baseline (we excluded studies that focused exclusively on children or older adults); (4) data were collected prospectively from the first episode; (5) the outcome was a categorical definition of TRS, established using longitudinal prospective medication history; and (6) a non-TRS comparison group was recruited and followed up in the same manner as the TRS group. Studies were excluded if (1) they were clinical trials, or if non-antipsychotic treatments, such as CBT or ECT, were administered as part of the study procedure; (2) the study focussed exclusively on early or late-onset schizophrenia; or (3) inferential statistics measuring the association between baseline variables and TRS were not reported, and our subsequent requests to the authors for unpublished data were unsuccessful.

### Defining TRS

Only recently has attention been given to the standardisation of TRS criteria (Farooq *et al*., 2013; Suzuki *et al*., 2012; Lee *et al*., 2015; Howes *et al*., 2017); therefore, we did not restrict studies to one definition of TRS. We did, however, only include studies with a categorical definition of TRS to capture the key underlying concept-at least two treatment failures – and differentiate TRS from relative measures of response/nonresponse. If patients took clozapine at follow-up, we inferred that they met criteria for TRS. Clozapine prescription is likely to underestimate the true proportion of patients with TRS (Howes *et al*., 2012), but it is a pragmatic criterion, since clozapine is only used for TRS, except in very rare indications (e.g. psychosis in the context of Parkinson's disease or for people who suffer severe side-effects to other antipsychotics).

### Literature search

Studies were identified by searching Pubmed, PsychINFO (up to October 2017), Medline (up to October 2017), Embase (up to October 2017), and OpenGrey on the 1 November 2017. In addition, we examined the first 20 pages of Google Scholar using terms ‘predictor AND treatment-resistant AND schizophrenia’ on 3 January 2018. No restrictions were placed on the publication date, but searches were restricted to the titles and abstracts of papers (and subject headings in Medline, Embase, and PsychINFO), studies published in English, and studies using human participants. Search terms for Pubmed were as follows: ‘((treatment resistant) OR (treatment resistance) OR (treatment refractory)) AND (schizophrenia) AND ((longitudinal) OR (prospective))’. Search strategies for other databases can be found in Appendix 1. We screened the title and abstracts of all identified studies and then performed full-text screening of all potentially eligible studies. Potentially eligible studies were cross-referenced; additional relevant studies were identified by hand-searches of the references, and by screening papers which had previously cited these studies. Each additional paper was also hand-searched until no new studies were identified. When full-text articles were not available, the corresponding author was contacted. Author SES conducted the initial screening, with APK independently screening the studies identified through database searches and all studies identified through cross-referencing.

### Quality assessment

We followed the PRISMA guidelines for reporting systematic reviews (Liberati *et al*., 2009).

Study quality was assessed using the Newcastle-Ottawa Scale (NOS) for cohort studies (http://www.ohri.ca/programs/clinical_epidemiology/oxford.asp). Eight items measure the selection, comparability, and outcome of each study. These items were modified for this review, for example, follow-up needed to have been longer than one year to score on the item concerning adequate duration of follow-up (see Appendix 2). Authors SES and APK independently rated each study on the NOS (Appendix 3), any differences in rating were discussed between authors and final ratings were a consensus.

When available, we report adjusted hazard (HR) or odds ratios (OR) with 95% confidence intervals (95% CI) in parentheses, for predictors measured at baseline.

## Results

**Fig. 1.**
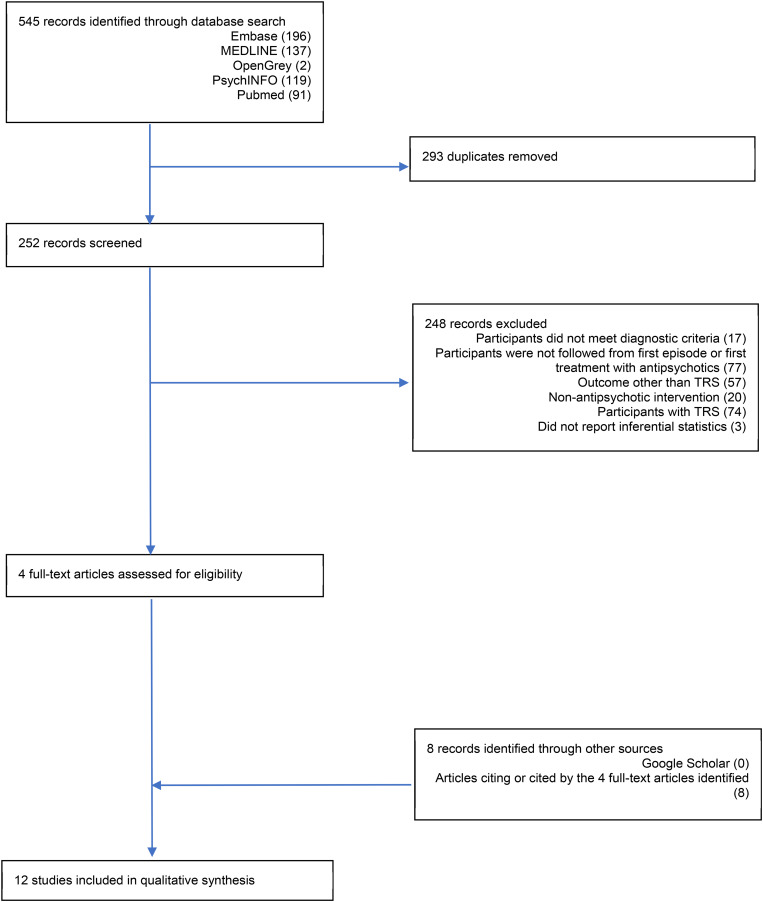
PRISMA flow diagram.

A total of 12 studies were identified for inclusion in this review. Study screening is depicted in Fig. 1 and a summary of the number of participants recruited into each study is presented in Table 1. Database searches identified 545 records, 293 of which were duplicates and removed. A total of 252 records were screened and 248 were excluded. The main reasons for exclusion were: the study did not follow participants from the first episode or first treatment with antipsychotics (31%), participants recruited after TRS had been identified (29%), and an outcome other than TRS was reported (23%). The remaining four records were examined in more detail, as were the eight records identified by cross-referencing. Only duplicates were identified through Google Scholar. Of the 12 studies, 11 were published in peer-reviewed academic journals. One study was unpublished (Chan *et al*., 2014), however, after corresponding with the authors, a full report was identified on the funding body's website containing enough information to be included in this review (https://rfs1.fhb.gov.hk/app/fundedsearch/projectdetail.xhtml?id=1363).

**Table 1.** The twelve studies included in this review, with details on the number of participants recruited and the length of follow-up

Study	Number of participants							Length of follow-up (years)
	Baseline	Follow-up	(%)	TRS	(%)	Non-TRS	(%)	
Chan *et al*. (2014)	469	469	(100)	160	(34)	309	(66)	16
Demjaha *et al*. (2017)	557	274	(49)	62	(23)	212	(77)	10
Horsdal *et al*. (2017*a*)*	390	390	(100)	52	(13)	338	(87)	2
Horsdal *et al*. (2017*b*)*	3252	3252	(100)	359	(11)	2893	(89)	2
Kim *et al*. (2017)	114 749	NR		NR		NR		NR
Lally *et al*. (2016)	283	240	(85)	81	(34)	159	(66)	5
Meltzer *et al*. (1997)	322	322	(100)	196	(61)	126	(39)	4
Sorensen *et al*. (2014)	5968	5328	(89)	1223	(23)	4105	(77)	34
Üçok *et al*. (2016)	187	105	(56)	28	(27)	77	(73)	2
Wimberley *et al*. (2016*a*)*	13 349	13 349	(100)	2313	(17)	11 036	(83)	17
Wimberley *et al*. (2016*b*)	9332	8044	(86)	1703	(21)	6341	(79)	14
Wimberley *et al*. (2017)*	862	862	(100)	181	(21)	681	(79)	11

NB: NR, not reported; *, analysis of data also presented in Wimberley *et al*. (2016*b*)

Of the 12 included studies, eight presented original data and four presented data on additional exposures within the same cohort as a previous study, or a subset thereof. Of these eight, three were population cohort studies. Both Sorensen *et al*. (2014) and Wimberley *et al*. (2016b) used Danish population registers: data was extracted from multiple national databases and linked using a unique personal identification number. Additional analyses of Wimberley *et al*. (2016b)'s data tested whether urbanicity (Wimberley *et al*., 2016*a*), the polygenic risk score for schizophrenia (PRS-SZ; Wimberley *et al*., 2017), functioning (Horsdal *et al*., 2017*b*), and C-reactive protein levels (Horsdal *et al*., 2017*a*) could predict TRS. The third population cohort came from South Korea (Kim *et al*., 2017). The remaining five studies analysed longitudinal first episode psychosis patient cohorts (Meltzer *et al*., 1997; Chan *et al*., 2014; Lally *et al*., 2016; Üçok *et al*., 2016; Demjaha *et al*., 2017).

In population registries, a proxy definition of first-episode psychosis is required. In the Danish studies, the first International Classification of Diseases (ICD; World Health Organization, 1993) diagnosis of schizophrenia was used to define the baseline cohort. The South Korean study used ICD diagnosis of schizophrenia and the first use of antipsychotics to define the baseline cohort. When using diagnoses, the first episode is likely to be later in the disease course, when compared to cohort studies. Additional study characteristics, including information about recruitment, diagnoses, and criteria for TRS and non-TRS can be found in Appendix 4. The variables measured, and tested as predictors of TRS, varied considerably across studies, therefore this information is summarised in Table 2. Appendix 5 contains the unadjusted and adjusted OR/HR, when these were reported.

**Table 2.** The variables which have been tested as predictors of TRS in the twelve studies included in this review

	Chan *et al*. (2014)	Demjaha *et al*. (2017)	Kim *et al*. (2017)	Lally *et al*. (2016)	Meltzer *et al*. (1997)	Sorensen *et al*. (2014)	Üçok *et al*. (2016)	Wimberley *et al*. (2016*b*)
Younger age of onset								
Alcohol misuse during follow-up period								
Antipsychotic polypharmacy during follow-up period								
C-reactive protein								
Comorbid diagnosis of personality disorder								
Comorbid diagnosis of suicide attempts								
Schizophrenia diagnosis								
Paranoid schizophrenia diagnosis								
Duration of first episode								
Longer DUP								
Early parental loss								
Lower education qualification								
Fewer years in education								
Employment status								
Black ethnicity								
Family history of schizophrenia								
Worse functioning								
Worse premorbid functioning								
Male								
Living arrangements								
Living arrangements during follow-up period								
Marital status								
Mode of onset								
Paternal age								
Polygenic risk score for schizophrenia								
Relapse despite adherence								
Relapse in first 6 months								
More relapses in the first three years								
Born in Autumn/winter								
Substance misuse								
Substance misuse during follow-up period								
More symptoms of psychosis								
Inpatient								
Urbanicity at birth								
Living in a more rural area at diagnosis								
Violent offence								
	Chan *et al*. (2014)	Demjaha *et al*. (2017)	Kim *et al*. (2017)	Lally *et al*. (2016)	Meltzer *et al*. (1997)	Sorensen *et al*. (2014)	Üçok *et al*. (2016)	Wimberley *et al*. (2016*b*)

NB: grey squares, variables not significantly associated with TRS; dark grey squares, variables significantly associated with TRS; all analyses using the Wimberley *et al*., Danish dataset were grouped under Wimberley *et al*. (2016*b*)

### Predictors of TRS

Chan *et al*. (2014) analysed a subsample of a first episode cohort who presented to mental health services over a five-year period and used clozapine prescription as a definition of TRS. As this was a case-control study including all patients with TRS and a ratio of two non-TRS patients for every TRS patient, the prevalence of TRS could not be calculated. The two groups were matched on baseline diagnosis. Chan *et al*. (2014) included age of onset, duration of untreated psychosis (DUP; days), duration of first episode, years of education, Premorbid Adjustment Scale (PAS) adult (19 + years) subscale score (Cannon-Spoor *et al*., 1982), substance misuse history, and the number of relapses in the first three years, in a Cox proportional hazard regression. The model significantly predicted TRS (Chi-square = 66.11, df = 7, *p* = <0.0001). While number of relapses in the first three years significantly predicted TRS, the only baseline predictors significantly associated with TRS were younger age of onset (HR = 0.88, 95% CI = 0.83–0.94) and poorer premorbid functioning (indicated by higher scores) according to the PAS (HR = 3.22, 95% CI = 1.43–7.23).

Demjaha *et al*. (2017) analysed data from the AESOP study, which recruited first episode patients over a three-year period and followed them up ten years later. The researchers entered gender, diagnosis, age of onset, negative symptoms, mode of onset, DUP (weeks), and ethnicity into a multivariate penalised logistic regression. The model selected five variables that predicted TRS: a diagnosis of schizophrenia at baseline (instead of psychotic depression; OR = 0.41, or psychotic mania; OR = 0.52), younger age of onset (years, OR = 0.97), higher severity of negative symptoms (OR = 1.09), an insidious mode of onset (instead of acute; OR = 1.28), and longer DUP (OR = 1.0013). Goodness-of-fit was measured using McFadden's pseudo *R*^2^ and correct classification rates were measured using the Brier score. A McFadden's pseudo *R*^2^ between 0.20 and 0.40 is considered a good model fit. The Brier score is used to evaluate predictive models; if the incidence of TRS is 23%, as estimated from Demjaha *et al*. (2017), a Brier score of 0 would be a perfect model while a score of 0.177 would be a non-informative model (Steyerberg *et al*., 2010). Demjaha *et al*. (2017) reported a McFadden's pseudo *R*^2^ of 0.10 and a Brier score of 0.146, suggesting that their model is not a good fit of the data nor is it a good classifier of TRS.

Kim *et al*. (2017), in their South Korean population cohort, estimated the cumulative incidence of clozapine use using the Kaplan–Meier method and log-rank test. They reported that younger age of onset predicted TRS. Unlike Chan *et al*. (2014) and Demjaha *et al*. (2017), Kim *et al*. (2017) examined age of onset categorically: defining younger age of onset as those aged between 15–20 years of age, and comparing them to a middle-onset group (21–44 years of age) and a late-onset group (45–64 years of age). Kim *et al*. (2017) also found, using the Walter–Elwood method (Walter and Elwood, 1975), a higher incidence of clozapine use, in those born during winter (December to February) when compared to those born in summer (June to August). This pattern remained true when stratifying season of birth by age of onset. Kim *et al*. (2017) reported no measures of overall model fit.

Lally *et al*. (2016) recruited first episode patients over a five-year period and used electronic medical records to follow them up five years later. They entered age of onset, Positive and Negative Symptom Scale (PANSS; Kay *et al*., 1987) scores, Global Assessment of Functioning (GAF; Hall, 1995) disability score, and GAF symptom scores into a penalised logistic regression, controlling for living arrangements, employment status, and alcohol/substance misuse during the follow-up period. Lally *et al*. (2016) included the PANSS total score, the positive, negative and general psychopathology subscale scores, as well as two individual items: lack of insight and conceptual disorganisation. None of the PANSS or GAF variables predicted TRS. Age at first contact with mental health services was split into four categories: 18–20, 21–25, 26–30, >31 years. Only age of onset between 18 and 20 years, compared to all other age groups, significantly predicted TRS (OR = 2.49, 95% CI = 1.25–4.94). The authors did not report the overall model fit. Age of onset was subsequently stratified by gender and ethnicity. Age of onset, between 18 and 20, only predicted TRS in males (OR = 2.13, 95% CI = 1.35–7.23) or those of black ethnicity (OR = 3.71, 95% CI = 1.44–9.56).

Meltzer *et al*. (1997) recruited patients at first admission to hospital for schizophrenia or schizoaffective disorder and followed them up for approximately four years. The authors examined the age of onset and gender in relation to TRS using a two-way analysis of variance (ANOVA). Gender was not associated with TRS but younger age of onset was. As males had a younger age of onset than females, the researchers examined the associations between age of onset and gender in more depth using simple effects ANOVA. In the non-TRS group, males had a younger age of onset (*F* = 6.6, df = 1, *p* < 0.01), however, in the TRS group, there was no difference in age of onset between males and females. Meltzer *et al*. (1997) calculated the conditional probability of a patient having TRS given their age of onset. For those aged between 15 and 18 years old, the probability of developing TRS was between 32% and 38% for both males and females.

Sorensen *et al*. (2014), in their Danish population cohort, entered a season of birth into a Cox proportion hazard regression adjusted for birth year and gender. The model did not significantly predict TRS. However, the authors found that being born in autumn (September to November), compared to spring (March to May), predicted TRS (HR = 1.24, 95% CI = 1.06–1.46). Unlike in Kim *et al*. (2017)'s study, being born in winter (December to February) failed to predict TRS.

Üçok *et al*. (2016) analysed a subsample of patients recruited into an ongoing first episode schizophrenia study. Üçok *et al*. (2016) entered the age of onset, DUP (days), first relapse despite adherence to antipsychotic treatment, relapse in the first six months, and antipsychotic polypharmacy during follow-up, into logistic regression. The authors did not report the overall model fit. Only first relapse despite adherence to antipsychotic treatment and antipsychotic polypharmacy predicted TRS. No baseline variables predicted TRS.

Wimberley *et al*. (2016*b*), in their Danish population cohort, entered twenty-three variables into a Cox proportion hazard regression. These variables included: gender, age at first schizophrenia diagnosis as a proxy for age of onset, family history of schizophrenia in first-degree relatives, winter birth (December to March), paternal age, parental loss before the age of 18, living alone, conviction for a violent offence before first schizophrenia diagnosis, level of education, employment status, urbanicity at first schizophrenia diagnosis, admission to psychiatric hospital before first schizophrenia diagnosis, schizophrenia subtype (paranoid *v.* all others), comorbid psychiatric diagnosis before first schizophrenia diagnosis, antipsychotic prescription in the year before first schizophrenia diagnosis, antidepressant prescription in the year before first schizophrenia diagnosis, and benzodiazepine prescription in the year before first schizophrenia diagnosis. Goodness-of-fit was measured using McFadden's pseudo *R*^2^ and correct classification rates using Harrell's C statistic; a C statistic of 0.5 would be a non-informative model while a score of 1 would be a perfect model. Wimberley *et al*. (2016*b*) report a McFadden's pseudo *R*^2^ of 0.027 and a Harrell's C statistic of 0.70, suggesting that this model is a good fit of the data and reasonable classifier of TRS. At baseline, younger age of onset (years, HR = 0.96, 95% CI = 0.95–0.97), living in less urban areas (rural *v.* capital area, HR = 1.44, 95% CI = 1.25–1.65), higher education (higher *v.* primary education, HR = 0.88, 95% CI = 0.79–0.98), psychiatric hospital admission at diagnosis (HR = 2.07, 95% CI = 1.87–2.29), having spent more than 30 bed-days in a psychiatric hospital in the year before diagnosis (HR = 1.54, 95% CI = 1.35–1.75), paranoid subtype diagnosis (HR = 1.24, 95% CI = 1.13–1.37), comorbid personality disorder (HR = 1.24, 95% CI = 1.11–1.39), comorbid suicide attempt (HR = 1.21, 95% CI = 1.07–1.39), antipsychotic use (HR = 1.51, 95% CI = 1.35–1.69), antidepressant use (HR = 1.15, 95% CI = 1.03–1.29), and benzodiazepines use (HR = 1.22, 95% CI = 1.10–1.37), all predicted TRS. Data on additional exposures within the same cohort, or a subset thereof, were published separately. Lower levels of urbanicity (Wimberley *et al*., 2016*a*) and severely impaired functioning (a GAF functioning score ⩽30; Horsdal *et al*., 2017*b*) predicted TRS, but the polygenic risk score for schizophrenia (PRS-SZ; Wimberley *et al*., 2017) and C-reactive protein levels (Horsdal *et al*., 2017*a*) did not predict TRS.

### Subcategories of TRS

Some patients have little or no response to antipsychotic treatment from the onset of their illness, while others initially respond to medication and then later develop TRS. Two of the studies in our review reported comparisons between subgroups TRS patients; early-onset TRS was operationalised as meeting criteria for TRS from the onset of schizophrenia and delayed-onset TRS as meeting criteria after a period of symptomatic remission. Chan *et al*. (2014) found no differences, in demographics, clinical characteristics, or premorbid functioning, between early-onset TRS (*N* = 17, 11.64%) and delayed-onset TRS (*N* = 129, 88.36%). Lally *et al*. (2016) found no differences in demographics between the two groups, but the early-onset TRS group (*N* = 56, 70%) had a younger mean age of onset than the delayed-onset TRS group (*N* = 24, 30%).

## Discussion

This review identified twelve research papers that examined predictors of TRS. Seven of the studies included in this review tested the age of onset as a predictor, and six reported that younger age of onset predicted TRS. Given that multiple definitions of the age of onset – age of onset of psychotic symptoms, age of first diagnosis of schizophrenia, age of first contact with mental health services – were reported and data was treated both continuously and categorically, this is a robust finding. Other potential risk factors, that have been identified by more than one study, include diagnosis, level of functioning, male gender, and season of birth.

A recent meta-analysis linked younger age of onset to multiple poor outcomes in schizophrenia: more hospitalisations, more negative symptoms, more relapses, poorer social/occupational functioning, and poorer global outcome (Immonen *et al*., 2017). Many of these poor outcomes have also been associated with TRS. Immonen *et al*. (2017) found that males had a younger age of onset and, therefore, samples with a higher proportion of males tended to show stronger associations between age of onset and outcomes. In the studies included in this review, the association between age of onset and TRS is unlikely to be wholly confounded by gender, as the proportion of males ranged from 49% (Kim *et al*., 2017) to 67% (Lally *et al*., 2016) and the studies which controlled for gender still showed an effect of age of onset (Meltzer *et al*., 1997; Lally *et al*., 2016; Wimberley *et al*., 2016*b*; Demjaha *et al*., 2017). In schizophrenia, age of onset has been thought to reflect genetic liability for the disease; younger age of onset has been associated with an increased familial risk of schizophrenia (Hilker *et al*., 2017; Byrne *et al*., 2018). Could, therefore, TRS be the result of increased genetic risk? While Wimberley *et al*. (2017) found no association between PRS-SZ and TRS, other work published by Frank *et al*. (2014) reports that an increased PRS-SZ is associated with TRS. In addition, rare copy number variations have been associated with both TRS (Martin and Mowry, 2015) and childhood-onset schizophrenia (Addington and Rapoport, 2009). Therefore, patients with TRS, who also have a younger age of onset, may have a more salient genetic influence than later-onset cases, although further work is required to substantiate this claim.

This review complements previous reviews by Gillespie *et al*. (2017) and Carbon and Correll (2014). Gillespie *et al*. (2017) examined studies comparing patients with treatment-resistant to patients with treatment-responsive schizophrenia. They included all study methodologies, but excluded studies where treatment-responsiveness was defined solely as not meeting treatment-resistant criteria. Carbon and Correll (2014) examined studies identifying predictors of response and remission. The researchers focused on first-episode psychosis studies where participants were followed up for five years. Some of the predictors of TRS, identified in this review, were found to be associated with less chance of response/remission by Carbon and Correll (2014), e.g. younger age of illness onset, poor premorbid adjustment, being male, lower level of education, living in a rural environment, diagnosis of schizophrenia, longer duration of untreated psychosis, poorer functioning, and worse psychopathology. However, Carbon and Correll (2014) also associated less chance of response/remission with being single, family history of psychosis, greater cognitive dysfunction, more family conflicts, and substance misuse; characteristics not identified as predictors of TRS. There was relatively little overlap between this review and Gillespie *et al*. (2017)'s review. In terms of studies included, only Meltzer *et al*. (1997)'s study was included in both reviews. In terms of characteristics associated with TRS, Gillespie *et al*. (2017) identified five neuroimaging studies, nine gene-association studies, and two studies of neurocognitive function, and these studies were not included in our review. The examination of biological markers, associated with TRS, within longitudinal study designs is rare; this is understandable for genome-wide association studies, which require large sample sizes more easily acquired using a cross-sectional methodology. However, there is a clear gap in the literature investigating biological markers that change over time (for example, proinflammatory cytokines or differently methylated positions within the epigenome) and TRS as an outcome. In terms of neuroimaging research, a review by Nakajima *et al*. (2015) found only five studies which compared patients with TRS to non-TRS patients, none of which had identified neural correlates of TRS. McGuire and Dazzan (2017) highlight only one study where neuroimaging data predicted a six-year, non-remitting course of illness. Longitudinal imaging studies of TRS are still relatively rare and constitute another gap in the literature.

Of the studies included in this review, few identified characteristics of abnormal neurodevelopment as predictors of TRS, despite neurodevelopment changes being linked with schizophrenia. The neurodevelopmental theory of schizophrenia proposes that disrupted normal development, in utero or early infancy, leads to deficits in psychophysiological and neurological functioning in childhood or early adolescence, and eventually to prodromal or diagnostic symptoms of schizophrenia (Jablensky *et al*., 2017; Murray *et al*., 2017). Previous research has linked characteristics of abnormal development with TRS; higher rates of minor physical anomalies (Lin *et al*., 2015), more neurological soft signs (de Bartolomeis *et al*., 2018), poor verbal intelligence and fluency (Kravariti *et al*., 2018), and poor verbal memory (Joober *et al*., 2002; de Bartolomeis *et al*., 2013). None of the studies in this review included variables measuring physiology during development or cognition at the first episode. Only Chan *et al*. (2014) examined premorbid functioning, retrospectively using the PAS. They found no difference, between the TRS and non-TRS groups, in terms of functioning during childhood, early adolescence, or late adolescent. There was a difference in functioning after the age of 19 and subsequently, worse functioning predicted TRS in their final model. If educational attainment can be considered a proxy for development only lower level of education qualification was found to significantly predict TRS (Wimberley *et al*., 2016*b*). The number of years in education was not predictive of TRS (Chan *et al*., 2014). Abnormal neurodevelopment and neuropsychology have not been sufficiently investigated as potential predictors of TRS.

Our review has illuminated some gaps in the existing literature, where potential predictors have not been fully investigated, however, we believe our review has captured all published work and identified predictors that, with further study, may prove to be clinically useful in determining treatment for patients with schizophrenia.

### Strengths and limitations

The main strength of this review is that we have focused solely on studies that included temporal forecasting (observations at baseline that are used to predict outcomes at follow-up), and as such eliminated recall bias and established a key component necessary for predictive models. All the studies included in this review are likely to be sufficiently powered to detect predictors of TRS. All the studies reported large sample sizes, and most followed participants for more than one year. Although no studies reported *a priori* power analysis, and only Meltzer *et al*. (1997) reported an *ad hoc* power analysis, we believe lack of power is unlikely to explain these results.

When attrition reduces the sample size at follow-up of longitudinal studies, consequently, statistical power is also reduced. For the studies we have reviewed, that reported on participants lost to follow-up, it is unlikely that the low attrition rates introduced bias. In particular, many studies used Cox proportional hazard regression; an analytic method that not only takes into account that individuals lost to follow-up may develop TRS, but also that individuals may develop TRS after the study endpoint. However, TRS, in particular, may be biased by attrition. There is a case both that TRS patients may be more likely to drop out of research studies due to their higher severity of symptoms and worse social and occupational functioning, and that responders are more likely to drop out as they lose touch with clinical services, but we are not aware of any published studies examining attrition in relation to treatment response.

One limitation to consider, when discussing the findings from these studies, is that some patients may have been misclassified. None of the studies included in this review explicitly accounted for adherence to medication, therefore characteristics may be predicting nonadherence rather than treatment resistance. None of the studies measured antipsychotic plasma levels, therefore characteristics may be predicting sub-therapeutic drug plasma levels, as a consequence of nonadherence, noncompliance, or pharmacokinetics, rather than treatment resistance. McCutcheon *et al*. (2015) found that 44% of patients referred to an outpatient service for clozapine treatment had sub-therapeutic conventional-antipsychotic plasma levels. On the other hand, it is unlikely that TRS patients have been wrongly classified as responders because the long follow-up periods allow plenty of time for a diagnosis of TRS to be established. Most studies had follow-ups longer than four years; the average delay before being treated for TRS estimated by Howes *et al*. (2012). The definitions of TRS, used in these studies, are pragmatic criteria: any predictors identified by these naturalistic studies are generalisable to real-world, clinical settings where adherence, compliance, or drug plasma levels influence treatment.

The use of multiple definitions of TRS is a problem across all TRS literature; Suzuki *et al*. (2011) reviewed 33 studies of prospective studies of pharmacological interventions for TRS and found that all 33 definitions of TRS were different. Howes *et al*. (2017) reviewed 42 clinical trials and found only two studies which used identical criteria. In addition, some studies use clozapine prescription as a proxy for TRS. When clozapine is under-prescribed, supposed predictors of TRS may, in fact, represent predictors of clozapine initiation (e.g. clinicians' attitudes towards clozapine prescription). All of the studies, identified in this review, used existing data, not designed to examine TRS, and researchers had to established proxy definitions based on the data available to them. When evidence concerning predictors of TRS is not consistent, it can be hard to draw a clear conclusion about the validity of the predictor, yet when the evidence is consistent across studies, with different definitions, the predictor in question is highly likely to generalise to other cohorts and have clinical validity.

Finally, we must consider the statistical methodology used to establish predictors. A common misconception is that predictive accuracy can be inferred from explanatory accuracy. However, the two are different and should be assessed separately (Shmueli, 2010). Only three studies included in this review reported the overall model fit, and only two reported statistics that measure the predictive validity of the model. Additionally, in predictive modelling, variable selection and overfitting must be considered. Lally *et al*. (2016) and Demjaha *et al*. (2017) attempted to reduce overfitting by penalising regression coefficients. However, none of the studies used holdout data (training data), cross-validation, or external validation to evaluate the predictive power of models; the latter being the current ‘gold-standard’ approach. In terms of variable selection, the only methods reported were LASSO regression (Demjaha *et al*., 2017) and step-wise selection using statistical significance (Chan *et al*., 2014; Üçok *et al*., 2016). Stepwise methods are no longer considered appropriate for explanatory models, but stepwise-type algorithms are very useful in predictive modelling (Shmueli, 2010), as long as the selection criteria rely on predictive power (e.g. Akaike information criterion) rather than explanatory power (e.g. statistical significance), as was the case in these studies. These methodological limitations must be taken into consideration when evaluating predictive models. The studies included in the review, on the whole, report analyses designed to identify explanatory variables of TRS. Future studies will need to use more robust prediction methods before moving from statistical prediction to clinical prediction.

## Conclusion

The aim of this systemic literature review was to identify predictors of treatment-resistant schizophrenia from prospective longitudinal studies. In choosing to focus exclusively on longitudinal studies, we have filled a gap in the existing literature, and hope that consolidating this information will be of use to researchers attempting to identify clinical predictors of TRS and the biological mechanisms causing TRS. We have identified earlier age of schizophrenia-onset as a robust predictor of TRS, with evidence that male gender, autumn/winter birth, poor premorbid functioning and rural upbringing may also contribute. We have also highlighted gaps in the literature namely, studies examining neuroimaging, immune, and genetic markers of TRS. Examination of biological markers, particularly within the framework of a prospective longitudinal study, has the potential to go beyond simple prediction and add to our understanding of the underlying causes of TRS. In conclusion, while early identification of TRS is clinically important, we currently have very limited knowledge of its predictors.

## Supplementary material

For supplementary material accompanying this paper visit https://doi.org/10.1017/S0033291719002083. click here to view supplementary material
