# Supplementary material for: Predictors of treatment resistant schizophrenia: a systematic review of prospective observational studies
Source: Psychol Med. 2019 Aug 29;51(1):44–53. doi: 10.1017/S0033291719002083 (PMC7856410; doi:10.1017/S0033291719002083)
Supplement: Supplementary file 1 [file S0033291719002083sup001.docx]

# Predictors of Treatment Resistant Schizophrenia: A systematic review of prospective observational studies

Smart S.E.^1^

Kępińska A.P.^1^

Murray R.M.^1^

MacCabe J.H.^1^

^1^Department of Psychosis Studies, Institute of Psychiatry, Psychology, & Neuroscience, King’s College London, 16 de Crespigny Park, London, SE5 8AF.

Contents

[Predictors of Treatment Resistant Schizophrenia: A systematic review of prospective observational studies 1](#_Toc10551673)

[Supplementary Material 1](#_Toc10551674)

[Appendix 1 1](#_Toc10551675)

[Appendix 2 4](#_Toc10551676)

[Appendix 2 5](#_Toc10551677)

[Appendix 4 10](#_Toc10551678)

[Appendix 5 16](#_Toc10551679)

[Appendix 6 20](#_Toc10551680)

[References 23](#_Toc10551681)

Table of Figures

[Table 1. *Modified Newcastle-Ottawa (NOS) scale for cohort studies* 4](#_Toc10551636)

[Table 2. *Modified Newcastle-Ottawa (NOS) scale ratings for the studies included in this review.* 5](#_Toc10551637)

[Table 3. *Study Characteristics.* 10](#_Toc10551638)

[Table 4. *Unadjusted and adjusted OR/HR, when reported,* *for the studies included in this review* 16](#_Toc10551639)

[Table 5. PRISMA Checklist 20](#_Toc10551640)

# Supplementary Material

### Appendix 1

On the 1^st^ November 2017 we ran the following searches:

Pubmed:

((treatment resistant[Title/Abstract]) OR (treatment resistance[Title/Abstract]) OR (treatment refractory[Title/Abstract])) AND (schizophrenia[Title/Abstract]) AND ((longitudinal[Title/Abstract]) OR (prospective[Title/Abstract]))

Restricted to Humans and English Language

N=91 (After removing duplicates N=73)

Embase 1974 to 2017 Week 44

((treatment resistant.mp.) OR (treatment resistance.mp.) OR (treatment refractory.mp.)) AND (schizophrenia.mp.) AND ((longitudinal.mp.) OR (prospective.mp.))

Restricted to Abstracts and Humans and English Language

N=196 (After removing duplicates N=93)

Ovid MEDLINE(R) 1946 to October Week 4 2017

((treatment resistant.mp.) OR (treatment resistance.mp.) OR (treatment refractory.mp.)) AND (schizophrenia.mp.) AND ((longitudinal.mp.) OR (prospective.mp.))

Restricted to Abstracts and Humans and English Language

N=137 (After removing duplicates N=32)

PsycINFO 1806 to October Week 4 2017

((treatment resistant.mp.) OR (treatment resistance.mp.) OR (treatment refractory.mp.)) AND (schizophrenia.mp.) AND ((longitudinal.mp.) OR (prospective.mp.))

Restricted to Abstracts and Humans

N=119 (After removing duplicates N=55)

OpenGrey

((treatment resistant) OR (treatment resistance) OR (treatment refractory)) AND (schizophrenia) AND ((longitudinal) OR (prospective))

N=2 (After removing duplicates N=2)

In addition, we examined the first 20 pages of google scholar using the terms ‘predictor AND treatment resistant AND schizophrenia’ on 3rd January 2018.

<https://scholar.google.co.uk/scholar?hl=en&as_sdt=0%2C5&q=predictor+AND+treatment+resistant+AND+schizophrenia&btnG>

| Appendix 2 Table 1. *Modified Newcastle-Ottawa (NOS) scale for cohort studies* | | | | | | | |
| --- | --- | --- | --- | --- | --- | --- | --- |
| **Selection** | | | | **Comparability** | **Outcome** | | |
| **Representativeness of the cohort who developed the outcome (max 1)** | **Selection of the cohort who did not develop the outcome (max 1)** | **Ascertainment of exposure (max 1)** | **Demonstration that outcome of interest was not present at start of study (i.e. when predictors were measured) (max 1)** | **Comparability of cohorts on the basis of the design or analysis (max 2)** | **Assessment**  **of outcome (max 1)** | **Was follow-up long enough for outcomes to occur (max 1)** | **Adequacy of follow up of cohorts (max 1)** |
| a) Truly representative of the average patient with psychosis in the community *  b) Somewhat representative of the average patient with psychosis in the community *  c) Selected group of patients with psychosis  d) No description of the derivation of the cohort | a) Drawn from the same community as the exposed cohort *  b) Drawn from a different source  c) No description of the derivation of the non-exposed cohort | a) Secure record (e.g. clinical records) *  b) Structured interview *  c) Written self-report  d) No description | a) Yes *  b) No | a) Study controls for baseline variables correlated with the outcome *  b) Study controls for any additional  factors/other sensitivity analyses * | a) Independent blind assessment *  b) Record linkage *  c) Self report  d) No description | a) Yes (1 year or longer) *  b) No | a) Complete follow up; all subjects accounted for *  b) Subjects lost to follow up unlikely to introduce bias; small number lost; description of those lost *  c) Follow up rate is large; no description of those lost  d) No statement |
| *, Criteria which equate to 1 point | | | | | | | |

| Appendix 2 Table 2. *Modified Newcastle-Ottawa (NOS) scale ratings for the studies included in this review.* | | | | | | | | | |
| --- | --- | --- | --- | --- | --- | --- | --- | --- | --- |
| **Study** | **Representiveness of the TRS cohort** | **Selection of the Non-TRS cohort** | **Ascertainment of exposure** | **Demonstration that outcome of interest was not present at start of study** | **Comparability of cohorts on the basis of the design/analysis (max 2)** | **Assessment of outcome** | **Was follow-up long enough for outcome to occur** | **Adequacy of follow up of cohorts** | **Score (max 9)** |
| Chan *et al.* (2014) | a) Truly representative of the average patient with psychosis in the community * | a) Drawn from the same community as the exposed cohort * | a) Secure record (e.g. clinical records) * | a) Yes * | b) Study controls for any additional  factors/other sensitivity analyses * | b) Record linkage * | a) Yes (1 year or longer) * | d) No statement | 7 |
| Demjaha *et al.* (2017) | a) Truly representative of the average patient with psychosis in the community * | a) Drawn from the same community as the exposed cohort * | a) Secure record (e.g. clinical records) *  AND  b) Structured interview * | a) Yes * | b) Study controls for any additional  factors/other sensitivity analyses * | b) Record linkage * | a) Yes (1 year or longer) * | b) Subjects lost to follow up unlikely to introduce bias; small number lost; description of those lost * | 8 |
| Horsdal *et al.* (2017a) | b) Somewhat representative of the average patient with psychosis in the community * | a) Drawn from the same community as the exposed cohort * | a) Secure record (e.g. clinical records) * | b) No | b) Study controls for any additional  factors/other sensitivity analyses * | b) Record linkage * | a) Yes (1 year or longer) * | d) No statement | 6 |
| Horsdal *et al.* (2017b) | b) Somewhat representative of the average patient with psychosis in the community * | a) Drawn from the same community as the exposed cohort * | a) Secure record (e.g. clinical records) * | a) Yes * | b) Study controls for any additional  factors/other sensitivity analyses * | b) Record linkage * | a) Yes (1 year or longer) * | b) Subjects lost to follow up unlikely to introduce bias; small number lost; description of those lost * | 8 |
| Kim *et al.* (2017) | a) Truly representative of the average patient with psychosis in the community * | a) Drawn from the same community as the exposed cohort * | a) Secure record (e.g. clinical records) * | a) Yes * | b) Study controls for any additional  factors/other sensitivity analyses * | b) Record linkage * | Not reported | d) No statement | 6 |
| Lally *et al.* (2016) | a) Truly representative of the average patient with psychosis in the community * | a) Drawn from the same community as the exposed cohort * | a) Secure record (e.g. clinical records) *  AND  b) Structured interview * | a) Yes * | a) Study controls for baseline variables correlated with the outcome * | b) Record linkage * | a) Yes (1 year or longer) * | b) Subjects lost to follow up unlikely to introduce bias; small number lost; description of those lost * | 8 |
| Meltzer *et al.* (1997) | c) Selected group of patients with psychosis | a) Drawn from the same community as the exposed cohort * | a) Secure record (e.g. clinical records) *  AND  b) Structured interview * | b) No | b) Study controls for any additional  factors/other sensitivity analyses * | b) Record linkage * | Not reported | d) No statement | 4 |
| Sorensen *et al.* (2014) | a) Truly representative of the average patient with psychosis in the community * | a) Drawn from the same community as the exposed cohort * | a) Secure record (e.g. clinical records) * | a) Yes * | b) Study controls for any additional factors * | b) Record linkage * | a) Yes (1 year or longer) * | b) Subjects lost to follow up unlikely to introduce bias; small number lost; description of those lost * | 8 |
| Üçok *et al.* (2016) | c) Selected group of patients with psychosis | a) Drawn from the same community as the exposed cohort * | a) Secure record (e.g. clinical records) *  AND  b) Structured interview * | a) Yes * | a) Study controls for baseline variables correlated with the outcome* | b) Record linkage * | a) Yes (1 year or longer) * | b) Subjects lost to follow up unlikely to introduce bias; small number lost; description of those lost * | 7 |
| Wimberley *et al.* (2016a) | a) Truly representative of the average patient with psychosis in the community * | a) Drawn from the same community as the exposed cohort * | a) Secure record (e.g. clinical records) * | a) Yes * | a) Study controls for baseline variables correlated with the outcome *  AND  b) Study controls for any additional  factors/other sensitivity analyses * | b) Record linkage * | a) Yes (1 year or longer) * | b) Subjects lost to follow up unlikely to introduce bias; small number lost; description of those lost * | 9 |
| Wimberley *et al.* (2016b) | a) Truly representative of the average patient with psychosis in the community * | a) Drawn from the same community as the exposed cohort * | a) Secure record (e.g. clinical records) * | a) Yes * | a) Study controls for baseline variables correlated with the outcome *  AND  b) Study controls for any additional  factors/other sensitivity analyses * | b) Record linkage * | a) Yes (1 year or longer) * | b) Subjects lost to follow up unlikely to introduce bias; small number lost; description of those lost * | 9 |
| Wimberley *et al.* (2017) | b) Somewhat representative of the average patient with psychosis in the community * | a) Drawn from the same community as the exposed cohort * | a) Secure record (e.g. clinical records) * | a) Yes * | a) Study controls for baseline variables correlated with the outcome * and  b) Study controls for any additional factors * | b) Record linkage * | a) Yes (1 year or longer) * | d) No statement | 8 |

| Appendix 4 Table 3. *Study Characteristics.* | | | | | | |
| --- | --- | --- | --- | --- | --- | --- |
| **Study** | **Study Design, Year of Enrolment** | **N** | **Diagnostic Criteria** | **Follow up (N)** | **TRS Operational Criteria** | **Non-TRS Operational Criteria** |
| Chan *et al.* (2014) | First episode psychosis cohort recruited between Jan 1998 and Aug 2003 (Hong Kong); selected patients were prospectively followed up at interview and through electronic medical records (ratio of 1:2 TRS patients to non-TRS patients) | 1400 | Schizophrenia, schizoaffective disorder, acute and transient psychotic disorder, psychosis not otherwise specified | Approximately 10 years; up to May 2004 (N=474, 34%) | N=165 (35%)  (1) Clozapine prescription or (2) At least moderate severity (score >=5) on 1 or more items on the positive symptom subscale of PANSS and a score <59 on the SOFAS at follow up with no periods of symptomatic or functional improvement in the preceding 6 months after receiving >=2 different types of antipsychotic medications sequentially and each for >=4 consecutive weeks at chlorpromazine (CPZ) equivalent dose of >=400mg/day | N=309 (65%)  None |
| Demjaha *et al.* (2017) | First episode psychosis cohort recruited between Sep 1997 and Aug 2000 (UK); prospectively followed up at interview and through electronic medical records | 557 | ICD-10: F10-F29, F30-F33 | Approximately 10 years (N=274, 49%) | N=62 (23%)  (1) Persistent psychotic symptoms, defined as having a rating of at least moderate severity on one or more positive symptoms as rated by SCAN and despite recorded adherence to medication, after two sequential antipsychotic trials, each of at least 4 weeks’ duration at a daily dose of 400–600 mg of chlorpromazine equivalents | N=212 (77%)  (1) A period of at least 6 months’ duration in which no symptoms or only symptoms of mild severity, not interfering with daily functioning, were experienced |
| Horsdal *et al.* (2017a) | First diagnosis schizophrenia cohort curated from electronic medical records of patients born in Denmark after 1954, who had a baseline measure of C‐reactive protein (CRP), and whose first diagnosis of schizophrenia occurred between Feb 2000 and Nov 2012; prospectively followed up through electronic records | 390 | ICD-8: 295.x9 excluding 295.79 or ICD-10: F20 | 2 years; the follow up period ran from first diagnosis of schizophrenia until incidence of TRS, emigration, death, or 2 years after first schizophrenia diagnosis, whichever came first (N=390, 100%) | N=52 (13%)  (1) First clozapine prescription redemption or (2) Psychiatric hospital admission due to schizophrenia during antipsychotic treatment within the 18 months after having received two prior antipsychotic monotherapy trials of adequate duration (6 weeks); hospitalizations from the year prior to first diagnosis and until the study endpoint were included | N=338 (87%)  None |
| Horsdal *et al.* (2017b) | First diagnosis schizophrenia cohort curated from electronic medical records of patients born in Denmark after 1954, who had a baseline measure of the GAF-F, and whose first diagnosis of schizophrenia occurred between Jan 2004 and Dec 2010; prospectively followed up through electronic records | 3252 | ICD-10: F20 | 2 years; the follow up period ran from first diagnosis of schizophrenia until incidence of TRS, emigration, death, or 2 years after first schizophrenia diagnosis, whichever came first (N=3252, 100%) | N=359 (11%)  (1) First clozapine prescription redemption or (2) Psychiatric hospital admission due to schizophrenia during antipsychotic treatment within the 18 months after having received two prior antipsychotic monotherapy trials of adequate duration (6 weeks); hospitalizations from the year prior to first diagnosis and until the study endpoint were included | N=2893 (89%)  None |
| Kim *et al.* (2017) | New antipsychotic-users cohort curated from electronic medical records of patients between Jan 2008 and Dec 2014 (South Korea); prospectively followed up through electronic records | 114,749 | ICD-10: F20 | Not reported; for some patients follow up exceeded 6 years | N not reported  (1) First prescription of clozapine | N not reported  None |
| Lally *et al.* (2016) | First episode psychosis cohort recruited between Dec 2005 and Oct 2010 (UK); prospectively followed up through electronic medical records | 283 | ICD-10: F20.0, F25.0, F28.0, F29.0 | 5 years (N=240, 85%) | N=81 (34%)  (1) Treated with clozapine at any point during the follow-up period or (2) Little or no symptomatic improvement to two consecutive treatments with antipsychotic medications of adequate dose (400 mg chlorpromazine equivalence) and duration (at least 6 weeks) excluding those intolerant to antipsychotic medications or those who self-discontinued medication | N=159 (66%)  None |
| Meltzer *et al.* (1997) | First hospitalisation for schizophrenia cohort (USA), recruitment period not reported, prospectively followed up through electronic medical records | 322 | DSM-III-R; schizophrenia, schizoaffective disorder | Not reported; for some patients follow up exceeded 4 years | N= 196 (61%)    (1) Persistent moderate to severe delusions, hallucinations, or thought disorder, despite at least three trials of typical neuroleptic drugs for at least 6 weeks at adequate doses  or  (2) Pervasive negative symptoms, such as withdrawal, anhedonia,  poverty of thought content, a deficit in volition, and lack of  energy, despite at least three trials of typical neuroleptic drugs for at least 6 weeks at adequate doses | N= 126 (39%)    (1) Those who had at most mild positive and negative symptoms during  the most recent course of neuroleptic treatment |
| Sorensen *et al.* (2014) | First diagnosis schizophrenia cohort curated from electronic medical records of patients born in Denmark between 1950 and 1970 whose first diagnosis of schizophrenia occurred between 1975 and 1990; prospectively followed up through electronic records | 5968 | ICD-8: 295 | 5-34 years; follow up to first clozapine prescription, date of death, or 31st December 2009, whichever came first (N=5328, 89%) | N=1223 (23%)   (1) First prescription of clozapine between 1995 to 2009 | N=4105 (77%)  None |
| Üçok *et al.* (2016) | First episode psychosis cohort recruited between 1996 and 2016 (Turkey); prospectively followed up at interview | 187 | DSM-IV: schizophrenia | Not reported; minimum 6 months for TRS patients and 2 years for non-TRS patients (N=105, 56%) | N=28 (27%)  (1) Clozapine prescription | N=777 (3%)  None |
| Wimberley *et al.* (2016a) | First diagnosis schizophrenia cohort curated from electronic medical records of patients born in Denmark after 1955 and whose first diagnosis of schizophrenia occurred between Jan 1996 and Jul 2013; prospectively followed up through electronic records | 13,349 | ICD-10: F20 | Median of 7 (IQR: 3-12) years; individuals were followed from their first diagnosis of schizophrenia until they met criteria for TRS, emigrated from Denmark, died, or until 1st July 2013, whichever came first (N=13,349, 100%) | N=2313 (17%)  (1) Clozapine prescription or (2) Psychiatric hospital admission due to schizophrenia, with evidence of treatment adherence, after having received two prior antipsychotic monotherapy trials of adequate duration (6 weeks), counted from one year prior to the first recorded schizophrenia diagnosis | N=11,036 (83%)  None |
| Wimberley *et al.* (2016b) | First diagnosis schizophrenia cohort curated from electronic medical records of patients born in Denmark after 1955 and whose first diagnosis of schizophrenia occurred between Jan 1996 and Dec 2006; prospectively followed up through electronic records | 9332 | ICD-8: 295.x9 (excluding 295.79) or ICD-10: F20 | Median 9 (IQR: 6-12) years; individuals were followed from their first diagnosis of schizophrenia until emigration, death, or 31st Dec 2010, whichever came first (N=8044, 86%) | N=1703 (21%)  (1) Clozapine prescription or (2) Psychiatric hospital admission due to schizophrenia during antipsychotic treatment within the 18 months after having received two prior antipsychotic monotherapy trials of adequate duration (6 weeks), counted from one year prior to the first recorded schizophrenia diagnosis | N=6341 (79%)  None |
| Wimberley *et al.* (2017) | First diagnosis schizophrenia cohort curated from electronic medical records of patients born in Denmark after 1981, who had a DNA sample available, and whose first diagnosis of schizophrenia occurred between 1999 and 2007; prospectively followed up through electronic records | 862 | ICD-10: F20 | Median 5 (IQR: 4-7) years; individuals were followed from their first diagnosis of schizophrenia until incidence of TRS, emigration, death, or 31st Dec 2010, whichever came first (N=862, 100%) | N=181 (21%)   (1) Clozapine prescription or (2) Psychiatric hospital admission due to schizophrenia during antipsychotic treatment within the 18 months after having received two prior antipsychotic monotherapy trials of adequate duration (6 weeks), counted from one year prior to the first recorded schizophrenia diagnosis | N=681 (79%)  None |
| Abbreviations: DNA, deoxyribonucleic acid; DSM, Diagnostic Statistical Manual; GAF-F, Global Assessment of Functioning – functioning scale; ICD, International Classification of Diseases; IQR, interquartile range; PANSS, Positive and Negative Schizophrenia Symptom scale; SCAN, Schedules for Clinical Assessment in Neuropsychiatry, SCAN; SOFAS, Social and Occupational Functioning Assessment Scale; TRS, treatment resistant schizophrenia. | | | | | | |

| Appendix 5 Table 4. *Unadjusted and adjusted OR/HR, when reported,* *for the studies included in this review* | | | | | |
| --- | --- | --- | --- | --- | --- |
| **Predictors** | **Study** | **Unadjusted** | | **Adjusted** | |
|  |  | **OR/HR** | **95%CI** | **OR/HR** | **95%CI** |
| **Patient Characteristics** |  |  |  |  |  |
| Born Autumn (September–November) vs. spring (March–May) | Sorensen et al. (2014)✝ | NR | NR | 1.24 | 1.06–1.46 |
| Born December–March vs April–November | Wimberley *et al.* (2016b)✝ | NR | NR | 1.01 | 0.91–1.11 |
| Born Summer (June–August) vs. spring (March–May) | Sorensen et al. (2014)✝ | NR | NR | 1.15 | 0.98–1.35 |
| Born Winter (December–February) vs. spring (March–May) | Sorensen et al. (2014)✝ | NR | NR | 1.12 | 0.95–1.31 |
| CRP >3 mg/L vs. CRP 0–3 mg/L | Horsdal et al. (2017a)✝ | 1.14 | 0.67–1.96 | 0.99 | 0.56–1.73 |
| Early parental loss (<18 years) | Wimberley *et al.* (2016b)✝ | NR | NR | 0.97 | 0.81–1.16 |
| Education: primary vs higher education level | Wimberley *et al.* (2016b)✝ | NR | NR | 0.88 | 0.79–0.98 |
| Education: years of education | Chan *et al.* (2014)✝ | NR | NR | 0.98 | 0.92–1.04 |
| Employment: long–term disability benefit vs. in work | Wimberley *et al.* (2016b)✝ | NR | NR | 1.14 | 0.95–1.36 |
| Employment: outside working force vs. in work | Wimberley *et al.* (2016b)✝ | NR | NR | 1.01 | 0.90–1.13 |
| Employment: unemployed vs. in work | Wimberley *et al.* (2016b)✝ | NR | NR | 0.95 | 0.79–1.15 |
| Family history of schizophrenia vs no family history | Wimberley *et al.* (2016b)✝ | NR | NR | 1.00 | 0.83–1.21 |
| Female vs male | Wimberley *et al.* (2016b)✝ | NR | NR | 1.07 | 0.96–1.19 |
| Female vs. male | Demjaha *et al.* (2017) | 0.48 | 0.25–0.86 | NR | NR |
| Level of urbanicity (diagnosis): provincial areas vs. capital area | Wimberley et al. (2016a)✝ | NR | NR | 1.40 | 1.26–1.56 |
| Level of urbanicity (diagnosis): rural areas vs. capital area | Wimberley et al. (2016a)✝ | NR | NR | 1.56 | 1.39–1.76 |
| Living alone vs in a couple | Wimberley *et al.* (2016b)✝ | NR | NR | 1.00 | 0.91–1.11 |
| Non–white vs. white | Demjaha *et al.* (2017) | 1.26 | 0.72–2.24 | NR | NR |
| PAS adult (19+ years) subscale score | Chan *et al.* (2014)✝ | NR | NR | 3.22 | 1.43–7.23 |
| Paternal age | Wimberley *et al.* (2016b)✝ | NR | NR | 1.00 | 0.99–1.01 |
| Polygenic risk score | Wimberley et al. (2017)✝ | 1.09 | 0.92–1.30 | 1.13 | 0.95–1.35 |
| Urbancity: provincial area (>10 000 inhabitants) vs. capital area (capital and suburb to the capital) | Wimberley *et al.* (2016b)✝ | NR | NR | 1.38 | 1.23–1.56 |
| Urbancity: rural area vs. capital area (capital and suburb to the capital) | Wimberley *et al.* (2016b)✝ | NR | NR | 1.44 | 1.25–1.65 |
| Substance misuse history | Chan *et al.* (2014)✝ | NR | NR | 1.34 | 0.67–2.69 |
| Violent offence | Wimberley *et al.* (2016b)✝ | NR | NR | 1.04 | 0.89–1.23 |
| **Disease Characteristics** |  |  |  |  |  |
| >30 bed–days in psychiatric hospital in year before fi rst schizophrenia diagnosis vs. 0 bed–days | Wimberley *et al.* (2016b)✝ | NR | NR | 1.54 | 1.35–1.75 |
| 1–30 bed–days in psychiatric hospital in year before fi rst schizophrenia diagnosis vs. 0 bed–days | Wimberley *et al.* (2016b)✝ | NR | NR | 1.11 | 0.96–1.27 |
| Age at first schizophrenia diagnosis | Wimberley *et al.* (2016b)✝ | NR | NR | 0.96 | 0.95–0.97 |
| Age of onset | Chan *et al.* (2014)✝ | NR | NR | 0.88 | 0.83–0.94 |
| Age of onset | Demjaha *et al.* (2017) | 0.93 | 0.89–0.97 | 0.97 | NR |
| Age of onset | Üçok et al. (2016) | NR | NR | 1.018 | NR |
| Age of onset <20 years | Lally *et al.* (2016) | NR | NR | 2.49 | 1.25–4.94 |
| Age of onset >31 years | Lally *et al.* (2016) | NR | NR | 0.55 | 0.27–1.12 |
| Age of onset 21–25 years | Lally *et al.* (2016) | NR | NR | 0.70 | 0.37–1.32 |
| Age of onset 26–30 years | Lally *et al.* (2016) | NR | NR | 1.14 | 0.57–2.28 |
| Diagnosis: depressive psychosis vs. schizophrenia | Demjaha *et al.* (2017) | 0.15 | 0.02–0.50 | 0.52 | NR |
| Diagnosis: manic psychosis vs. schizophrenia | Demjaha *et al.* (2017) | 0.12 | 0.02–0.42 | 0.41 | NR |
| Diagnosis: Paranoid subtype | Wimberley *et al.* (2016b)✝ | NR | NR | 1.24 | 1.13–1.37 |
| Diagnosis: previous comorbid comorbid diagnosis of suicide attempt | Wimberley *et al.* (2016b)✝ | NR | NR | 1.21 | 1.07–1.39 |
| Diagnosis: previous comorbid comorbid diagnosis of other schizophrenia spectrum disorders | Wimberley *et al.* (2016b)✝ | NR | NR | 1.05 | 0.94–1.16 |
| Diagnosis: previous comorbid comorbid diagnosis of personality disorder | Wimberley *et al.* (2016b)✝ | NR | NR | 1.24 | 1.11–1.39 |
| Diagnosis: previous comorbid comorbid diagnosis of schizoaffective disorder | Wimberley *et al.* (2016b)✝ | NR | NR | 1.18 | 0.95–1.45 |
| Diagnosis: previous comorbid comorbid diagnosis of substance abuse | Wimberley *et al.* (2016b)✝ | NR | NR | 0.99 | 0.88–1.11 |
| Diagnosis: previous comorbid diagnosis of depression | Wimberley *et al.* (2016b)✝ | NR | NR | 1.11 | 0.97–1.26 |
| Duration of first episode | Chan *et al.* (2014)✝ | NR | NR | 1.003 | 1.001–1.004 |
| Duration of untreated psychosis (days) | Chan *et al.* (2014)✝ | NR | NR | 0.90 | 0.69–1.18 |
| Duration of untreated psychosis (weeks) | Demjaha *et al.* (2017) | 1.004 | 1.001–1.006 | 1.0013 | NR |
| Duration of untreated psychosis (days) | Üçok et al. (2016) | NR | NR | 1.03 | NR |
| GAF-F | Lally *et al.* (2016) | NR | NR | 0.98 | 0.96–1.01 |
| GAF-F score 1–30 vs. 31–100 | Horsdal et al. (2017b)✝ | 1.45 | 1.08–1.94 | 1.38 | 1.03–1.86 |
| GAF-S | Lally *et al.* (2016) | NR | NR | 0.97 | 0.94–1.00 |
| Inpatient at first schizophrenia diagnosis | Wimberley *et al.* (2016b)✝ | NR | NR | 2.07 | 1.87–2.29 |
| Mode of onset: insidious vs. acute | Demjaha *et al.* (2017) | 0.13 | 1.44–0.00 | 1.28 | NR |
| Negative symptoms (dervived using FA from the SCAN) | Demjaha *et al.* (2017) | 1.24 | 1.08–1.42 | 1.09 | NR |
| PANSS Conceptual disorganization | Lally *et al.* (2016) | NR | NR | 1.13 | 0.83–1.56 |
| PANSS Lack of judgement and insight | Lally *et al.* (2016) | NR | NR | 1.14 | 0.89–1.47 |
| PANSS Negative | Lally *et al.* (2016) | NR | NR | 1.03 | 0.96–1.11 |
| PANSS Positive | Lally *et al.* (2016) | NR | NR | 1.01 | 0.94–1.07 |
| PANSS Total | Lally *et al.* (2016) | NR | NR | 1.01 | 0.98–1.04 |
| Psychotropic medication redeemed in year before first schizophrenia diagnosis: antidepressants | Wimberley *et al.* (2016b)✝ | NR | NR | 1.15 | 1.03–1.29 |
| Psychotropic medication redeemed in year before first schizophrenia diagnosis: antipsychotics | Wimberley *et al.* (2016b)✝ | NR | NR | 1.51 | 1.35–1.69 |
| Psychotropic medication redeemed in year before first schizophrenia diagnosis: benzodiazepines | Wimberley *et al.* (2016b)✝ | NR | NR | 1.22 | 1.10–1.37 |
| **During Treatment** |  |  |  |  |  |
| Antipsychotic polypharmacy (prior to clozapine for clozapine users) | Üçok et al. (2016) | NR | NR | 0.15 | NR |
| Having a first relapse despite being adherent to non–clozapine antipsychotic treatment | Üçok et al. (2016) | NR | NR | 3.93 | NR |
| Number of relapses in the first three years | Chan *et al.* (2014)✝ | NR | NR | 1.45 | 1.18–1.78 |
| Relapse in first 6 months (prior to clozapine use for clozapine users) | Üçok et al. (2016) | NR | NR | 1.03 | NR |
| NB: Kim et al. (2017) and Meltzer et al. (1997) did not report OR/HR and therefore not included in this table.  Abbreviations: ✝, Hazard Ratios; CRP, C‐reactive protein; FA, factor analysis; GAF-F, Global Assessment of Functioning – functioning scale; GAF-S, Global Assessment of Functioning – symptoms scale; PANSS, Positive and Negative Schizophrenia Symptom scale; PAS, Premorbid Assessment Scale; SCAN, Schedules for Clinical Assessment in Neuropsychiatry. | | | | | |

| Appendix 6 Table 5. PRISMA Checklist | | | |
| --- | --- | --- | --- |
| **Section/topic** | **#** | **Checklist item** | **Reported on page #** |
| **TITLE** | | |  |
| Title | 1 | Identify the report as a systematic review, meta-analysis, or both. | 1 |
| **ABSTRACT** | | |  |
| Structured summary | 2 | Provide a structured summary including, as applicable: background; objectives; data sources; study eligibility criteria, participants, and interventions; study appraisal and synthesis methods; results; limitations; conclusions and implications of key findings; systematic review registration number. | 1 |
| **INTRODUCTION** | | |  |
| Rationale | 3 | Describe the rationale for the review in the context of what is already known. | 4 |
| Objectives | 4 | Provide an explicit statement of questions being addressed with reference to participants, interventions, comparisons, outcomes, and study design (PICOS). | 4-5 |
| **METHODS** | | |  |
| Protocol and registration | 5 | Indicate if a review protocol exists, if and where it can be accessed (e.g., Web address), and, if available, provide registration information including registration number. | No |
| Eligibility criteria | 6 | Specify study characteristics (e.g., PICOS, length of follow-up) and report characteristics (e.g., years considered, language, publication status) used as criteria for eligibility, giving rationale. | 4-5 |
| Information sources | 7 | Describe all information sources (e.g., databases with dates of coverage, contact with study authors to identify additional studies) in the search and date last searched. | 5 |
| Search | 8 | Present full electronic search strategy for at least one database, including any limits used, such that it could be repeated. | 5/Supplementary material |
| Study selection | 9 | State the process for selecting studies (i.e., screening, eligibility, included in systematic review, and, if applicable, included in the meta-analysis). | 5-6 |
| Data collection process | 10 | Describe method of data extraction from reports (e.g., piloted forms, independently, in duplicate) and any processes for obtaining and confirming data from investigators. | 5-6 |
| Data items | 11 | List and define all variables for which data were sought (e.g., PICOS, funding sources) and any assumptions and simplifications made. | 6 |
| Risk of bias in individual studies | 12 | Describe methods used for assessing risk of bias of individual studies (including specification of whether this was done at the study or outcome level), and how this information is to be used in any data synthesis. | 6/Supplementary material |
| Summary measures | 13 | State the principal summary measures (e.g., risk ratio, difference in means). | 6 |
| Synthesis of results | 14 | Describe the methods of handling data and combining results of studies, if done, including measures of consistency (e.g., I^2^) for each meta-analysis. | NA |

Page 1 of 2

| **Section/topic** | **#** | **Checklist item** | **Reported on page #** |
| --- | --- | --- | --- |
| Risk of bias across studies | 15 | Specify any assessment of risk of bias that may affect the cumulative evidence (e.g., publication bias, selective reporting within studies). | 14-17 |
| Additional analyses | 16 | Describe methods of additional analyses (e.g., sensitivity or subgroup analyses, meta-regression), if done, indicating which were pre-specified. | NA |
| **RESULTS** | | |  |
| Study selection | 17 | Give numbers of studies screened, assessed for eligibility, and included in the review, with reasons for exclusions at each stage, ideally with a flow diagram. | 6/Figure 1 |
| Study characteristics | 18 | For each study, present characteristics for which data were extracted (e.g., study size, PICOS, follow-up period) and provide the citations. | 6-11/Table 1/Table 2 |
| Risk of bias within studies | 19 | Present data on risk of bias of each study and, if available, any outcome level assessment (see item 12). | Supplementary material |
| Results of individual studies | 20 | For all outcomes considered (benefits or harms), present, for each study: (a) simple summary data for each intervention group (b) effect estimates and confidence intervals, ideally with a forest plot. | 6-11/Table 1/Table 2/ Supplementary material |
| Synthesis of results | 21 | Present results of each meta-analysis done, including confidence intervals and measures of consistency. | NA |
| Risk of bias across studies | 22 | Present results of any assessment of risk of bias across studies (see Item 15). | NA |
| Additional analysis | 23 | Give results of additional analyses, if done (e.g., sensitivity or subgroup analyses, meta-regression [see Item 16]). | NA |
| **DISCUSSION** | | |  |
| Summary of evidence | 24 | Summarize the main findings including the strength of evidence for each main outcome; consider their relevance to key groups (e.g., healthcare providers, users, and policy makers). | 11-16 |
| Limitations | 25 | Discuss limitations at study and outcome level (e.g., risk of bias), and at review-level (e.g., incomplete retrieval of identified research, reporting bias). | 14-16 |
| Conclusions | 26 | Provide a general interpretation of the results in the context of other evidence, and implications for future research. | 11-16 |
| **FUNDING** | | |  |
| Funding | 27 | Describe sources of funding for the systematic review and other support (e.g., supply of data); role of funders for the systematic review. | 1 |

*From:*  Moher D, Liberati A, Tetzlaff J, Altman DG, The PRISMA Group (2009). Preferred Reporting Items for Systematic Reviews and Meta-Analyses: The PRISMA Statement. PLoS Med 6(7): e1000097. doi:10.1371/journal.pmed1000097

For more information, visit: **www.prisma-statement.org**.

Page 2 of 2

###

### References

**Chan, S. K. W., Chang, W. C., Lee, E. H. M., Wong, G. H. Y., Hui, C. L. M., Chen, E. Y. H. & Sham, P. C.** (2014). Predictors of treatment resistant schizophrenia-spectrum disorder: 10-year retrospective study of first-episode psychosis. *Early Intervention in Psychiatry* **8**, 78.

**Demjaha, A., Lappin, J. M., Stahl, D., Patel, M. X., MacCabe, J. H., Howes, O. D., Heslin, M., Reininghaus, U. A., Donoghue, K., Lomas, B., Charalambides, M., Onyejiaka, A., Fearon, P., Jones, P., Doody, G., Morgan, C., Dazzan, P. & Murray, R. M.** (2017). Antipsychotic treatment resistance in first-episode psychosis: prevalence, subtypes and predictors. *Psychological Medicine* **47**, 1981-1989.

**Horsdal, H. T., Wimberley, T., Benros, M. E. & Gasse, C.** (2017a). C-reactive protein levels and treatment resistance in schizophrenia—A Danish population-based cohort study. *Human Psychopharmacology: Clinical and Experimental* **32**, e2632-n/a.

**Horsdal, H. T., Wimberley, T., Köhler-Forsberg, O., Baandrup, L. & Gasse, C.** (2017b). Association between global functioning at first schizophrenia diagnosis and treatment resistance. *Early Intervention in Psychiatry*, n/a-n/a.

**Kim, J. S., Park, C. M., Choi, J. A., Park, E., Tchoe, H. J., Choi, M., Suh, J. K., Kim, Y. H., Won, S. H., Chung, Y. C., Bae, K. Y., Lee, S. K., Park, S. C. & Lee, S. H.** (2017). The association between season of birth, age at onset, and clozapine use in schizophrenia. *Acta Psychiatrica Scandinavica* **136**, 445-454.

**Lally, J., Ajnakina, O., Di Forti, M., Trotta, A., Demjaha, A., Kolliakou, A., Mondelli, V., Reis Marques, T., Pariante, C., Dazzan, P., Shergil, S. S., Howes, O. D., David, A. S., MacCabe, J. H., Gaughran, F. & Murray, R. M.** (2016). Two distinct patterns of treatment resistance: clinical predictors of treatment resistance in first-episode schizophrenia spectrum psychoses. *Psychological Medicine* **46**, 3231-3240.

**Meltzer, H. Y., Rabinowitz, J., Lee, M. A., Cola, P. A., Ranjan, R., Findling, R. L. & Thompson, P. A.** (1997). Age at onset and gender of schizophrenic patients in relation to neuroleptic resistance. *Am J Psychiatry* **154**, 475-82.

**Sorensen, H. J., Foldager, L., Roge, R., Pristed, S. G., Andreasen, J. T. & Nielsen, J.** (2014). An association between autumn birth and clozapine treatment in patients with schizophrenia: a population-based analysis. *Nord J Psychiatry* **68**, 428-32.

**Üçok, A., Çıkrıkçılı, U., Ergül, C., Tabak, Ö., Salaj, A., Karabulut, S. & Correll, C. U.** (2016). Correlates of Clozapine Use after a First Episode of Schizophrenia: Results From a Long-term Prospective Study. *CNS Drugs* **30**, 997-1006.

**Wimberley, T., Gasse, C., Meier, S. M., Agerbo, E., MacCabe, J. H. & Horsdal, H. T.** (2017). Polygenic Risk Score for Schizophrenia and Treatment-Resistant Schizophrenia. *Schizophr Bull* **43**, 1064-1069.

**Wimberley, T., Pedersen, C. B., MacCabe, J. H., Støvring, H., Astrup, A., Sørensen, H. J., Horsdal, H. T., Mortensen, P. B. & Gasse, C.** (2016a). Inverse association between urbanicity and treatment resistance in schizophrenia. *Schizophrenia Research* **174**, 150-155.

**Wimberley, T., Støvring, H., Sørensen, H. J., Horsdal, H. T., MacCabe, J. H. & Gasse, C.** (2016b). Predictors of treatment resistance in patients with schizophrenia: a population-based cohort study. *The Lancet Psychiatry* **3**, 358-366.
